# Association of Low-Attenuation Noncalcified Plaque Shape and Degree of Intraplaque Embeddedness Using Atherosclerotic Imaging–enabled Quantitative CT with Acute Coronary Syndrome

**DOI:** 10.1148/ryct.250117

**Published:** 2026-07-23

**Authors:** Shant Malkasian, Nick S. Nurmohamed, Hyuk-Jae Chang, Andrew D. Choi, Hugo Marques, Sabee Molloi, Alexander van Rosendael, Benjamin J.W. Chow, Daniele Andreini, Edoardo Conte, Erica Maffei, Fabian Plank, Gianluca Pontone, Gudrun Feuchtner, Habib Samady, Hyung-Bok Park, Ibrahim Danad, Iksung Cho, Jeroen J. Bax, Hyun-Ji Lee, Ji Min Sung, Lohendran Baskaran, Martin Hadamitzky, Matthew J. Budoff, Peter H. Stone, Ran Heo, Ricardo C. Cury, Sang-Eun Lee, Subhi J. Al'Aref, Wijnand J. Stuijfzand, Yong-Jin Kim, Filippo Cademartiri, Renu Virmani, Jagat Narula, Kavitha Chinnaiyan, Todd C. Villines, Jonathon Leipsic, Leslee J. Shaw, Fay Y. Lin, Tami Crabtree, Udo Hoffman, James P. Earls

**Affiliations:** ^1^Department of Radiology, University of California, San Diego, 9452 Medical Ctr Dr, La Jolla, CA 92037; ^2^Vascular Medicine, Amsterdam UMC, Amsterdam, the Netherlands; ^3^Division of Cardiology, Severance Cardiovascular Hospital, Yonsei University College of Medicine, Seoul, Korea; ^4^Division of Cardiology and Department of Radiology, The George Washington University School of Medicine, Washington, DC; ^5^Hospital da Luz, Lisbon, Portugal; ^6^Department of Radiological Sciences, University of California, Irvine School of Medicine, Irvine, Calif; ^7^Department of Cardiology, Leiden University Medical Center, Leiden, the Netherlands; ^8^Division of Cardiology, University of Ottawa Heart Institute, Ottawa, Canada; ^9^Department of Cardiovascular Imaging, Centro Cardiologico Monzino, IRCCS, Milan, Italy; ^10^Department of Radiology, Area Vasta 1/ASUR Marche, Urbino, Italy; ^11^Department of Cardiology, Innsbruck Medical University, Innsbruck, Austria; ^12^Department of Radiology, Innsbruck Medical University, Innsbruck, Austria; ^13^Georgia Heart Institute, Gainesville, Ga; ^14^Department of Cardiology, International St Mary’s Hospital, Catholic Kwandong University College of Medicine, Incheon, Korea; ^15^Department of Cardiology, VU University Medical Center Amsterdam, Amsterdam, the Netherlands; ^16^Department of Cardiology, Leiden University Medical Center, Leiden, the Netherlands; ^17^National Heart Centre, Singapore; ^18^Department of Radiology and Nuclear Medicine, German Heart Center Munich, Munich, Germany; ^19^The Lundquist Institute, Harbor-UCLA Medical Center, Torrance, Calif; ^20^Division of Cardiovascular Medicine, Brigham and Women’s Hospital, Boston, Mass; ^21^Hanyang University Medical Center, Seoul, Korea; ^22^Department of Radiology, Baptist Cardiac and Vascular Institute, Coral Gables, Fla; ^23^Seoul St. Mary’s Hospital, Seoul, Korea; ^24^Department of Medicine, University of Arkansas for Medical Sciences, Little Rock, Ark; ^25^Seoul National University Hospital, Seoul, Korea; ^26^Department of Radiology, Fondazione Toscana Gabriele Monasterio/CNR, Pisa, Italy; ^27^CVPath Institute, Gaithersburg, Md; ^28^University of Texas Health, Houston, Tex; ^29^Department of Cardiology, William Beaumont Hospital, Royal Oak, Mich; ^30^Department of Cardiology, University of Virginia Health System, Charlottesville, Va; ^31^Department of Radiology, St. Paul’s Hospital, University of British Columbia, Vancouver, Canada; ^32^Department of Medicine and Population Health Sciences, Icahn School of Medicine at Mount Sinai, New York, NY; ^33^Cleerly Inc, Denver, Colo; ^34^Department of Radiology, The George Washington University School of Medicine, Washington, DC

**Keywords:** Coronary Angiography, Coronary Arteries

## Abstract

**Purpose:**

To assess the association of low-attenuation noncalcified plaque (LAP) morphologic features, including shape and degree of intraplaque embeddedness, using atherosclerotic imaging–enabled quantitative CT with acute coronary syndrome (ACS).

**Materials and Methods:**

In this secondary analysis of the Incident Coronary Syndromes Identified by CT (ICONIC) study, a retrospective-nested, case-control, multicenter study of patients with future ACS after coronary CT angiography propensity matched with controls, atherosclerotic imaging–enabled quantitative CT analysis was performed between February and September 2022. LAP morphology was determined qualitatively according to geometric shape (crescent, lobular, spherical, or bean) and degree of intraplaque embeddedness (<90°, 90°–179°, 180°–269°, 270°–360°) within the vessel wall. Shapes were based on visual assessment of LAP contours, including features of curvature, symmetry, and lobulated and protruding components. Adverse LAP morphology (ALM) was defined by morphologic features associated with ACS using log-rank testing. Multivariable Cox regression was performed with ALM as the independent variable, adjusting for plaque burden and diameter stenosis.

**Results:**

A total of 446 patients (mean age ± SD, 62.44 years ± 11.05; 277 male; 223 cases, 223 controls) with a mean follow-up of 2.45 years ± 2.49 were included in this study. Twenty-two patients were excluded due to missing images or poor image quality. Lobular, bean, and spherical LAP shape or LAP with a degree of intraplaque embeddedness greater than or equal to 180° was associated with increased ACS risk (log-rank *P* < .05 for each). These morphologic features thus defined ALM. Among 74 of 446 (16.6%) patients with ALM, 57 patients experienced ACS. Patients with ALM had a 3.24-fold increased risk for ACS (adjusted hazard ratio, 3.24 [95% CI: 1.44, 7.30]; *P* = .005).

**Conclusion:**

ALM was independently associated with ACS.

**Keywords:** Coronary Angiography, Coronary Arteries

ClinicalTrials.gov identifier: NCT02959099

*Supplemental material is available for this article.*

© The Author(s) 2026. Published by the Radiological Society of North America under a CC BY 4.0 license.

## Graphical abstract

**Figure:**
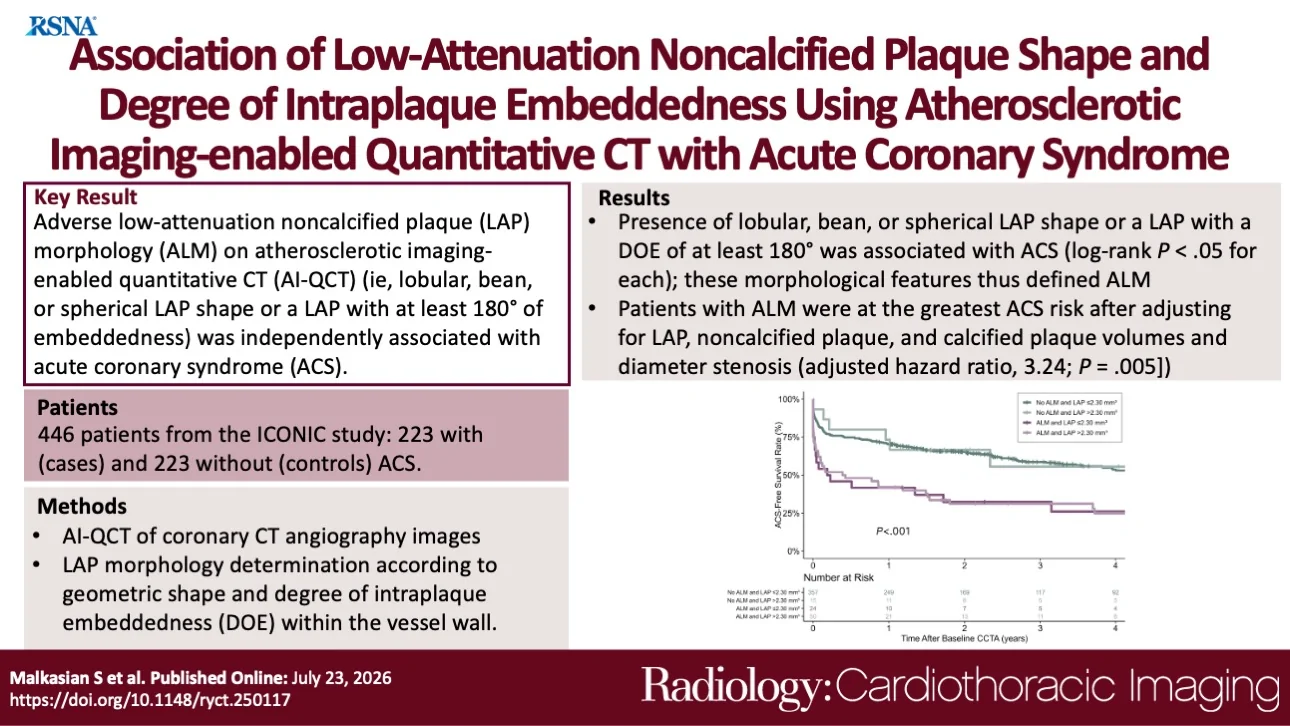

**Summary**

Adverse low-attenuation noncalcified plaque morphology using atherosclerotic imaging-enabled quantitative CT (ie, lobular, bean, or spherical low-attenuation noncalcified plaque shape or low-attenuation noncalcified plaque with ≥ 180° of embeddedness) was independently associated with acute coronary syndrome.

**Key Points**

■ In this secondary analysis of the Incident Coronary Syndromes Identified by CT study (*n* = 446), the presence of lobular, bean, or spherical low-attenuation noncalcified plaque (LAP) shape or a LAP with embeddedness greater than or equal to 180° at atherosclerotic imaging–enabled quantitative CT was associated with acute coronary syndrome (log-rank *P* <.05 for each); these morphologic features thus defined adverse LAP morphology.
■ Patients with adverse LAP morphology were at the greatest acute coronary syndrome risk after adjusting for LAP, noncalcified plaque, and calcified plaque volumes and diameter stenosis (adjusted hazard ratio, 3.24; *P* = .005).
■ Adverse LAP morphology presence incrementally improved acute coronary syndrome prediction compared with noncalcified and calcified plaque burden and diameter stenosis (area under the receiver operating characteristic curve, 0.70 vs 0.66; *P* = .02).

## Introduction

Identifying patients at high risk for acute coronary syndrome (ACS) remains one of the most challenging tasks in current atherosclerotic cardiovascular disease prevention practice. Despite the abundance of atherosclerotic cardiovascular disease risk-lowering strategies, ACS rates in both primary and secondary prevention remain high (1,2). Coronary CT angiography (CCTA) enables noninvasive assessment of atherosclerotic plaque characteristics and coronary artery stenosis. Conventionally, CCTA analysis focused on identifying obstructive coronary stenosis. However, in the Incident Coronary Syndromes Identified by CT (ICONIC) study, a multicenter, international, nested, case-control study comparing patients who underwent CCTA with subsequent ACS with patients who did not experience ACS, most culprit lesion precursors to ACS were nonobstructive. The findings of the ICONIC study, and several other studies, suggest that plaque burden quantification and characterization may be key to future ACS prognostication (3–5). Several large-scale studies have shown that the presence of high-risk plaque features, such as low-attenuation plaque and positive remodeling, confer increased risk for ACS (6–8), although the utility of these features has been limited by low positive predictive value. Moreover, the napkin-ring sign (9) and more recent radiomics study findings (10) suggest that plaque morphology may improve the stratification of patients with increased risk for ACS.

Although the correspondence of the napkin-ring sign with necrotic cores has been well studied via histopathologic examination (11), the geometric shape and degree of embeddedness of low-attenuation noncalcified plaque (LAP) has not been evaluated. Given the volumetric capability of CCTA, coupled with accurate and reproducible quantitative software, additional two-dimensional and three-dimensional characteristics of LAP can now be routinely evaluated. Therefore, the aim of this secondary analysis of the ICONIC study was to evaluate the independent association of the three-dimensional geometric shape and two-dimensional degree of intraplaque embeddedness (DOE) of LAP with ACS, quantified via atherosclerotic-based quantitative CT (AI-QCT), beyond that of LAP burden.

## Materials and Methods

### Study Patient Population

**Figure 1:**
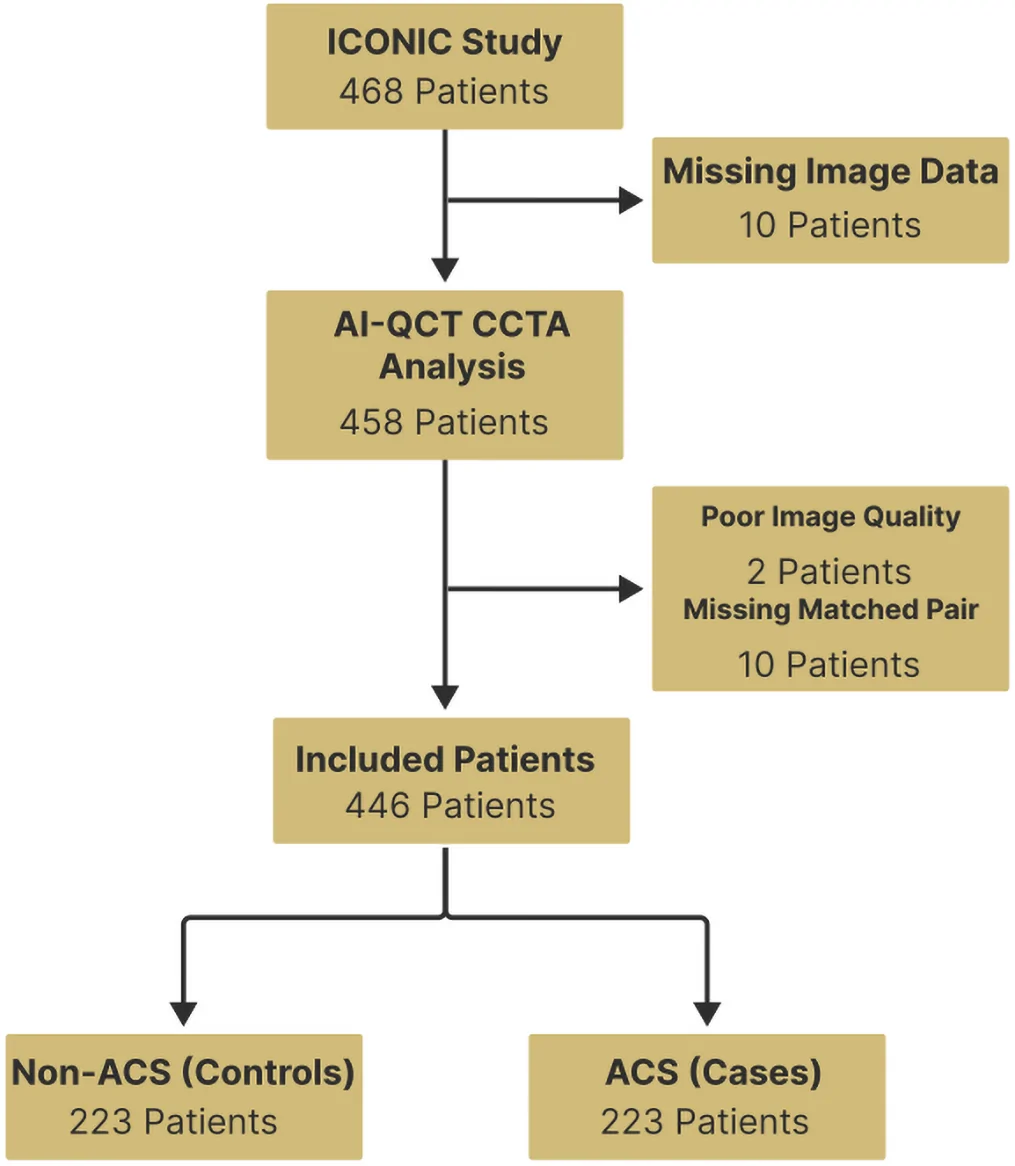
Flow chart shows study design. For this secondary analysis of the retrospective Incident Coronary Syndromes Identified by CT (ICONIC) study of the 468 patients, 10 were excluded because of missing image data. Of the remaining 458 patients, 12 were further excluded because of poor image quality or missing matched pairs, leaving 446 included patients, 223 patients with acute coronary syndrome (ACS) propensity matched to 223 controls. AI-QCT = atherosclerotic-imaging enabled quantitative CT, CCTA = coronary CT angiography.

In this study, AI-QCT was applied to assess LAP morphology in a secondary analysis of the multicenter international ICONIC study (NCT02959099) (5,12–16). This secondary analysis was conducted on data from a previously approved retrospective study. Institutional review board approval was obtained, along with a waiver of the requirement to obtain patient informed consent, and all data were handled in accordance with Health Insurance Portability and Accountability Act regulations. The ICONIC study was a nested case-control study of 234 patients who underwent CCTA before ACS during follow-up, and these patients were matched 1:1 to nonevent control patients who also underwent CCTA. Cases and controls were selected from the Coronary CT Angiography Evaluation for Clinical Outcomes: An International Multicenter (CONFIRM) registry of 25 416 patients who underwent baseline CCTA between October 2004 and August 2015 (17). The ICONIC study specifically included patients with no known history of coronary artery disease, defined as prior revascularization or myocardial infarction. Propensity scoring was used to match cases and controls according to clinical risk factors for coronary artery disease, including age, sex, self-reported ethnicity, history of hypertension and hyperlipidemia, family history of premature coronary artery disease, and smoking history, as well as one-, two-, or three-vessel coronary artery disease as assessed at CCTA. For this secondary analysis conducted between February and September 2022, 446 of 468 patients (95.30%; 223 controls, 223 cases) from the ICONIC study were included. Among the 22 patients who were excluded, 10 patients were excluded because of missing or corrupted CCTA images during the secondary analysis, and two patients were excluded because of insufficient image quality. The 10 corresponding matched pairs of these excluded patients were also excluded (Fig 1).

### ACS Adjudication

The method of ACS adjudication was previously published (5). Briefly, the primary outcome was ACS, including ST-elevation myocardial infarction, non–ST-elevation myocardial infarction, and unstable angina. ACS adjudication was based on cardiac enzyme measurements, electrocardiograms, and invasive coronary angiography, with the investigators (Appendix S1) blinded to CCTA data and using definitions established by the World Health Organization (18,19). In a subgroup of patients who experienced ACS, culprit lesion adjudication was also performed (Appendix S1).

### Quantitative CCTA Analysis and AI-QCT

All CCTA imaging was performed with greater than or equal to 64-detector row systems and in accordance with the Society of Cardiac Computed Tomography guidelines (20). CCTA images underwent AI-QCT analysis with a validated software service (version 2.3; Cleerly Laboratories) (21–23). Coronary vessels with a diameter greater than or equal to 1.5 mm were analyzed and segmented according to the 18-segment Society of Cardiac CT model (20). The presence of atherosclerosis was defined as any tissue structure within at least 1 mm^3^ of the coronary artery wall, differentiated from epicardial fat, epicardial tissue, or the coronary lumen. According to Hounsfield unit ranges, plaque volume was categorized as LAP (<30 HU), noncalcified plaque (30–350 HU), and calcified plaque (>350 HU) (24–26). Percent atheroma volume was calculated as total plaque volume ÷ coronary vessel volume × 100%. Percent LAP burden, noncalcified plaque burden, and calcified plaque burden were calculated as the specific plaque volume ÷ total plaque volume. Diameter stenosis was measured with a previously described AI-QCT method (21). AI-QCT plaque quantification has been previously validated against intravascular US imaging (23). Cardiac CT–trained technicians with at least 1 year of experience reviewed the AI-QCT results for all cases and performed manual adjustments when necessary, blinded to ACS outcomes.

A threshold of LAP greater than 2.30 mm^3^ was used to define lesions with substantial LAP burden. This decision was based on prior work demonstrating that this cut point showed the strongest association with lipid-rich plaque identified at intravascular US with near-infrared spectroscopy (23).

### LAP Morphology Evaluation

**Figure 2:**
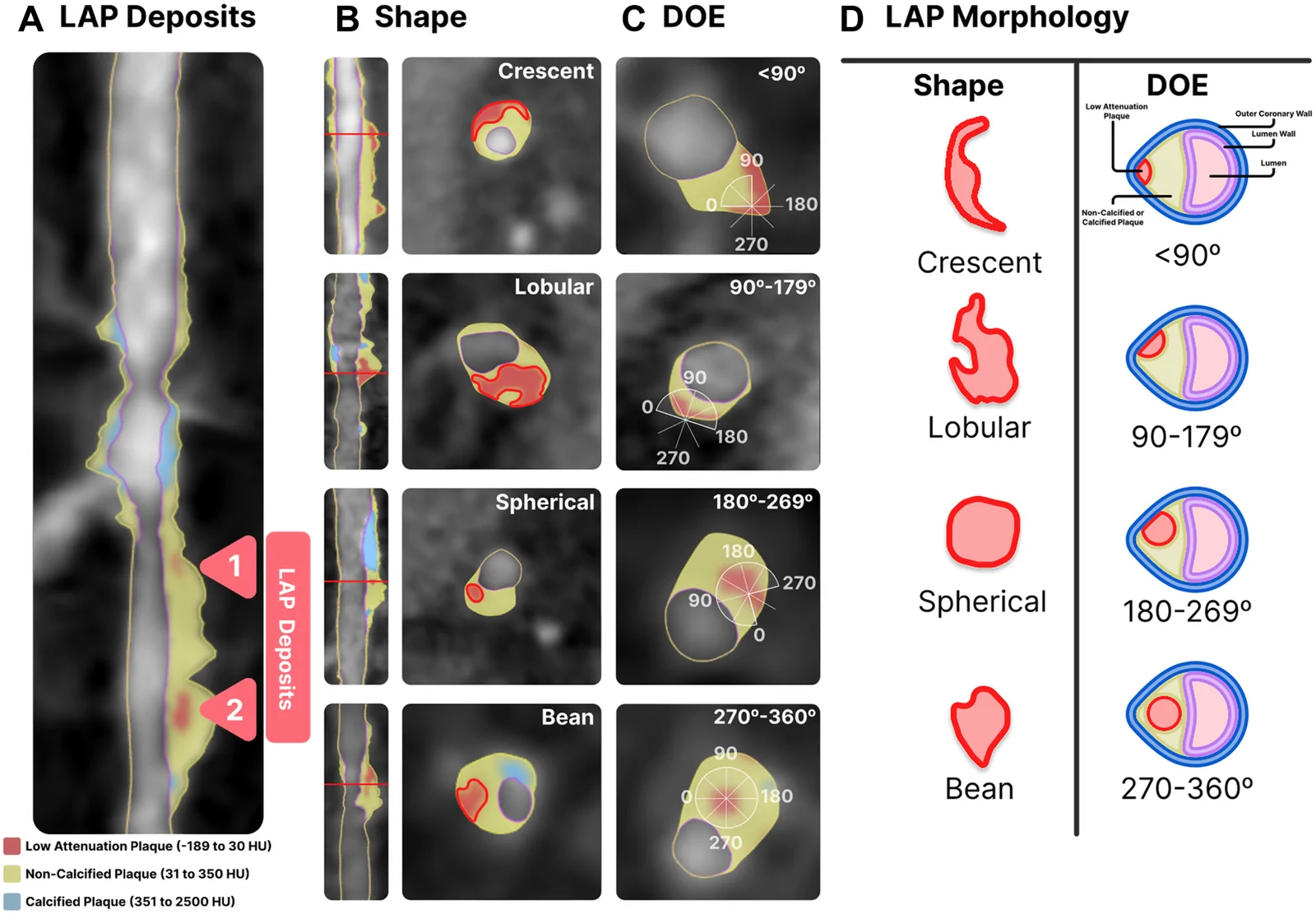
Images show the assessment of low-attenuation noncalcified plaque morphology at coronary CT angiography. Low-attenuation noncalcified plaque (LAP) morphology was assessed noninvasively via coronary CT angiography. **(A)** LAP morphology was assessed on the basis of the qualitative appearance of noncontiguous LAP deposits on straightened multiplanar reformatted images. LAP morphology was evaluated on the basis of **(B)** shapes in three-dimensional (crescent, lobular, spherical, or bean) and **(C)** degree of embeddedness (DOE) in the coronary wall on the cross-sectional section with the most LAP area (<90°, 90°–179°, 180°–269°, and 270°–360°). **(D)** Schematics show each morphologic feature.

LAP morphology of noncontiguous LAP deposits was qualitatively assessed by two board-certified level 3 CCTA readers in consensus agreement (J.P.E. and A.D.C., with 28 and 9 years of experience in cardiac imaging, respectively) at the LAP deposit-level. Readers were blinded to ACS outcomes. Readers jointly reviewed LAP morphology and directly reconciled differences at the time of review. LAP morphology was not assessed in smaller LAP deposits where the longest axis measurement was less than 0.50 mm or for patients with LAP greater than 0.50 mm^3^. LAP morphology was visualized with straightened multiplanar reformatted images with a colored plaque overlay tool, as available in AI-QCT (Fig 2A). LAP shape was categorized as crescent, lobular, bean, or spherical according to its three-dimensional appearance on cross-sectional and longitudinal straightened multiplanar reformatted images (Fig 2B). LAP shapes were classified according to visual assessment of plaque contours, including features such as curvature, symmetry, and the presence of lobulated or protruding components (Table S1). LAP shapes (eg, crescent, lobular, bean, and spherical) were defined, in part, heuristically based on the napkin-ring sign and the appearance of a necrotic core at histopathologic examination. The DOE of LAP was assessed according to the extent to which the LAP was circumscribed by surrounding noncalcified or calcified plaque (Fig 2C, 2D). On the cross-sectional image with the greatest LAP area within a lesion, DOE was determined in two dimensions as less than 90°, 90°–179°, 180°–269°, or 270°–360°. DOE was defined according to the premise that more embedded plaque represented a true necrotic core. LAP morphology was aggregated from the deposit level to the lesion and patient level for statistical analysis. Thus, multiple LAP shapes or DOE categories could be present in each patient. ACS event-free survival curve and log-rank test analysis were used to identify adverse LAP morphology (ALM).

### Statistical Analysis

All statistical analysis was performed by S. Malkasian. Mixed logistic regression was used to calculate odds ratios, using the pair-matched identifier as a random effect. Accuracy, sensitivity, specificity, positive predictive value, and negative predictive value were calculated to evaluate the diagnostic performance of LAP shape and DOE in identifying patients with ACS. For all measurements, exact binomial 95% CIs were calculated. Interobserver agreement on LAP shape and DOE and ALM was assessed by calculating the Cohen κ (<0.20 indicates slight agreement, 0.21–0.40 indicates fair agreement, 0.41–0.60 indicates moderate agreement, 0.61–0.80 indicates substantial agreement, and >0.80 indicates almost perfect agreement). The two experienced readers, blinded to each other’s assessments, independently evaluated LAP shape and DOE in a balanced subset of 12 randomly selected lesions.

ACS event-free survival curve analysis was conducted with the log-rank test to determine statistical significance. Univariable and multivariable Cox regression analyses were performed with the time to first ACS event or the event-free follow-up date. The robust variance estimator was used to adjust for matched pairs. Cox regression analysis was stratified by matched pairs. Multivariable Cox regression was performed using ALM as the independent variable, adjusting for LAP volume, noncalcified plaque volume, calcified plaque volume, and diameter stenosis. The concordance index, Akaike information criterion, and 2-year time-dependent area under the receiver operator characteristic curve (AUC) were used to assess multivariable model performance. AUC values were calculated and compared with the timeROC R package (R Development Core Team). For all patients, follow-up for ACS began after CCTA imaging. Right censoring was applied in patients without ACS occurrence during follow-up at the latest date of contact.

All statistical analyses were performed with R ( version 4.2.0; R Development Core Team). A *P* value less than .05 was considered statistically significant. Continuous variables are presented as means ± SDs or medians with IQRs depending on the distribution normality and compared using the Wilcoxon rank sum test. Categorical variables are presented as the numbers with percentages and compared using Pearson χ^2^ test or Fisher exact test when expected counts were less than or equal to 5. The Shapiro-Wilk test was used to determine distribution normality for continuous variables.

## Results

### Patient Characteristics

Of the 446 patients (mean age ± SD, 62.44 years ± 11.05; 277 male and 169 female), 223 patients experienced ACS during follow-up: ST-elevation myocardial infarction, 35 (15.7%); non–ST-elevation myocardial infarction, 111 (49.8%); unstable angina, 72 (32.0%); and ACS of unknown category, five (2.2%). The mean follow-up time was 2.45 years ± 2.49. The mean age of patients with ACS was 62.35 years ± 11.58 and for matched nonevent controls was 62.53 years ± 10.52 (*P *= .965). The number of male patients with ACS was 140 of 223 (62.78%) versus 137 of 223 (61.43%) for nonevent controls (*P* = .77) (Table 1).

**Table 1:** Patient Demographics

Demographic	Overall (*n* = 446)	ACS (Cases) (*n* = 223)	Non-ACS (Controls) (*n* = 223)	*P* Value
Age (y)	62.44 ± 11.05 (24–93)	62.35 ± 11.58 (24–93)	62.53 ± 10.52 (24–87)	.96
Follow-up time (days)	893.15 ± 909.55	396.65 ± 627.94	1389.65 ± 876.78	<.001
Male sex	277/446 (62.11)	140/223 (62.78)	137/223 (61.43)	.77
Female sex	169/446 (37.89)	83/223 (37.22)	86/223 (38.57)	.77
Family history of CAD	175/439 (39.86)	90/217 (41.47)	85/222 (38.29)	.50
Diabetes mellitus	116/446 (26.01)	44/223 (19.73)	72/223 (32.29)	.003
Hypercholesterolemia	239/444 (53.83)	124/221 (56.11)	115/223 (51.57)	.34
Hypertension	278/444 (62.61)	142/221 (64.25)	136/223 (60.99)	.48
Never smoker	169/354 (47.74)	82/185 (44.32)	87/169 (51.48)	.18
Former tobacco use	138/325 (42.46)	77/170 (45.29)	61/155 (39.35)	.28
Current tobacco use	117/444 (26.35)	66/222 (29.73)	51/222 (22.97)	.11
Ethnicity				.89
African	15/446 (3.36)	6/223 (2.69)	9/223 (4.04)	
White	206/446 (46.19)	104/223 (46.64)	102/223 (45.74)	
East Asian	104/446 (23.32)	52/223 (23.32)	52/223 (23.32)	
Latin American	2/446 (0.45)	1/223 (0.45)	1/223 (0.45)	
Middle Eastern	1/446 (0.22)	1/223 (0.45)	0/223 (0.00)	
Other or mixed	4/446 (0.90)	1/223 (0.45)	3/223 (1.35)	
South Asian	4/446 (0.90)	3/223 (1.35)	1/223 (0.45)	
Unknown	110/446 (24.66)	55/223 (24.66)	55/223 (24.66)	

Note.—Age is shown as mean ± SD with the range in parentheses. Continuous variables are reported as means ± SDs and compared using the Wilcoxon rank sum test. Categorical variables are reported as proportions with percentages in parentheses and compared using the Pearson χ^2^ test or the Fisher exact test. ACS = acute coronary syndrome, CAD = coronary artery disease.

### Defining ALM

Lobular, bean, and spherical LAP shapes were more frequent in ACS cases than controls, whereas crescent LAP shapes were not (lobular: 31 of 223 [13.9%] vs 13 of 223 [5.8%], *P* = .005; bean: 43 of 223 [19.3%] vs 11 of 223 [4.9%], *P* < .001; spherical: 15 of 223 [6.7%] vs 0 of 223 [0%]; crescent: 29 of 223 [13.0%] vs 21 of 223 [9.4%], *P* = .23). LAP with 90°–179°, 180°–269°, and 270°–360° DOE occurred more frequently in ACS cases than controls (90°–179°: 41 of 223 [18.4%] vs 25 of 223 [11.2%], *P* = .03; 180°–269°: 43 of 223 [19.3%] vs 10 of 223 [4.5%], *P* < .001; 270°–360°: 21 of 223 [9.4%] vs four of 223 [1.8%], *P* = .002), whereas less than 90° for DOE LAP was infrequent, with no evidence of a difference in its occurrence between groups (five of 223 [2.2%] vs four of 223 [1.8%], *P* = .74) (Table S2).

In the ACS-free survival curve analysis, lobular, bean, and spherical LAP shapes were associated with ACS (log-rank *P* = .001, *P* < .001, and *P* < .001, respectively), whereas crescent LAP shape was not (log-rank *P* = .52). LAPs with DOEs of 180°–269° and 270°–360° were significantly associated with ACS based on ACS-free survival curve analysis (log-rank *P* < .001 and *P* < .001, respectively), whereas LAPs with DOEs of less than 90° and 90°–179° were not (log-rank *P* = .49 and *P* = .05, respectively) (Figs S1 and S2). Thus, based on survival analysis, ALM was defined as the presence of one of the following morphologic features: bean, spherical, or lobular LAP shape or a LAP with a DOE of greater than or equal to 180°.

### Interobserver Agreement of ALM

There was substantial agreement in the identification of ALM between readers (J.P.E. and A.D.C.; κ = 0.67) (Figs S3, S4).

### Association of ALM with ACS

There were 70 patients of 446 (15.7%) with a lesion with LAP greater than 2.30 mm^3^, of whom 50 patients experienced ACS. Patients with a LAP greater than 2.30 mm^3^ had a 2.8-fold increased odds of ACS (odds ratio, 2.79 [95% CI: 1.58, 4.94]; *P *<.001). There were 74 patients of 446 (16.6%) with ALM, of whom 57 patients experienced ACS. Patients with ALM had a 4.2-fold increased odds of ACS (odds ratio, 4.16 [95% CI: 2.33, 7.42]; *P* < .001). The proportion of patients with no ALM and LAP less than or equal to 2.30 mm^3^, no ALM and a lesion with LAP greater than 2.30 mm^3^, ALM and LAP less than or equal to 2.30 mm^3^, and ALM and a lesion with LAP greater than 2.30 mm^3^ among patients with ACS was 159 of 223 (71.3%), seven of 223 (3.1%), 18 of 223 (8.1%), and 39 of 223 (17.5%), respectively (Table 2 and Table S2). The sensitivity, specificity, positive predictive value, negative predictive value, and accuracy of the presence of ALM in identifying patients with ACS was 26% (57 of 223 [95% CI: 20, 32]), 92% (206 of 223 [95% CI: 88, 95]), 77% (57 of 74 [95% CI: 66, 85]), 55% (206 of 372 [95% CI: 50, 60]), and 59% (263 of 446 [95% CI: 54, 63]), respectively (Table S3A). The lesion level association of ALM with development into culprit ACS-causing lesions was also assessed (Appendix S2; Tables S3B and S4; Fig S5).

**Table 2:** Atherosclerotic Plaque Characteristics from Coronary CT Angiography and Association with ACS

Characteristic	Overall (*n* = 446)	ACS (Cases) (*n* = 223)	Non-ACS (Controls) (*n* = 223)	Univariable OR	*P* Value
Total plaque volume (mm^3^)	227.40 (101.90, 448.10)	275.60 (121.20, 459.90)	199.20 (82.00, 427.70)	1.00 (1.00, 1.00)	.12
Low-attenuation noncalcified plaque volume (mm^3^)	0.00 (0.00, 0.40)	0.00 (0.00, 1.40)	0.00 (0.00, 0.10)	1.09 (1.02, 1.16)	.007
Noncalcified plaque volume (mm^3^)	148.85 (77.40, 280.70)	172.10 (83.90, 305.60)	127.90 (69.10, 235.20)	1.00 (1.00, 1.00)	.010
Total calcified plaque volume (mm^3^)	51.15 (8.10, 155.90)	58.70 (8.10, 161.60)	48.70 (8.10, 151.00)	1.00 (1.00, 1.00)	.86
Minimum lumen diameter (mm)	1.73 (1.41, 2.07)	1.69 (1.30, 2.03)	1.80 (1.43, 2.13)	0.70 (0.49, 1.00)	.05
Maximum remodeling index	1.40 (1.30, 1.60)	1.40 (1.30, 1.60)	1.40 (1.30, 1.60)	2.13 (0.94, 4.79)	.07
Maximum lesion length (mm)	40.25 (22.00, 66.75)	44.75 (23.50, 70.75)	38.13 (18.75, 63.00)	1.00 (1.00, 1.01)	.29
Maximum diameter stenosis (%)	37.00 (20.00, 52.00)	40.00 (23.00, 55.00)	31.00 (18.00, 47.00)	1.02 (1.01, 1.02)	<.001
Maximum lesion LAP > 2.30 mm^3^	70 (15.70)	50 (22.42)	20/223 (8.97)	2.79 (1.58, 4.94)	<.001
LAP > 0 mm^3^	157 (35.20)	95 (42.60)	62/223 (27.80)	1.93 (1.30, 2.86)	.001
ALM	74 (16.59)	57 (25.56)	17/223 (7.62)	4.16 (2.33, 7.42)	<.001
ALM and LAP					
No ALM and LAP ≤ 2.30 mm^3^	357 (80.04)	159 (71.30)	198 (88.79)	Ref.	NA
No ALM and lesion with LAP > 2.30 mm^3^	15 (3.36)	7 (3.14)	8 (3.59)	1.09 (0.39, 3.07)	.87
ALM and LAP ≤ 2.30 mm^3^	24 (5.38)	18 (8.07)	6 (2.69)	3.74 (1.45, 9.63)	.006
ALM and lesion with LAP > 2.30 mm^3^	50 (11.21)	39 (17.49)	11 (4.93)	4.42 (2.19, 8.90)	<.001

Note.—Data are numbers with percentages in parentheses or ORs with 95% CIs in parentheses. ACS = acute coronary syndrome, ALM = adverse low-attenuation noncalcified plaque morphology, LAP = low-attenuation noncalcified plaque, OR = odds ratio, Ref. = reference.

### Survival Analysis

**Figure 3:**
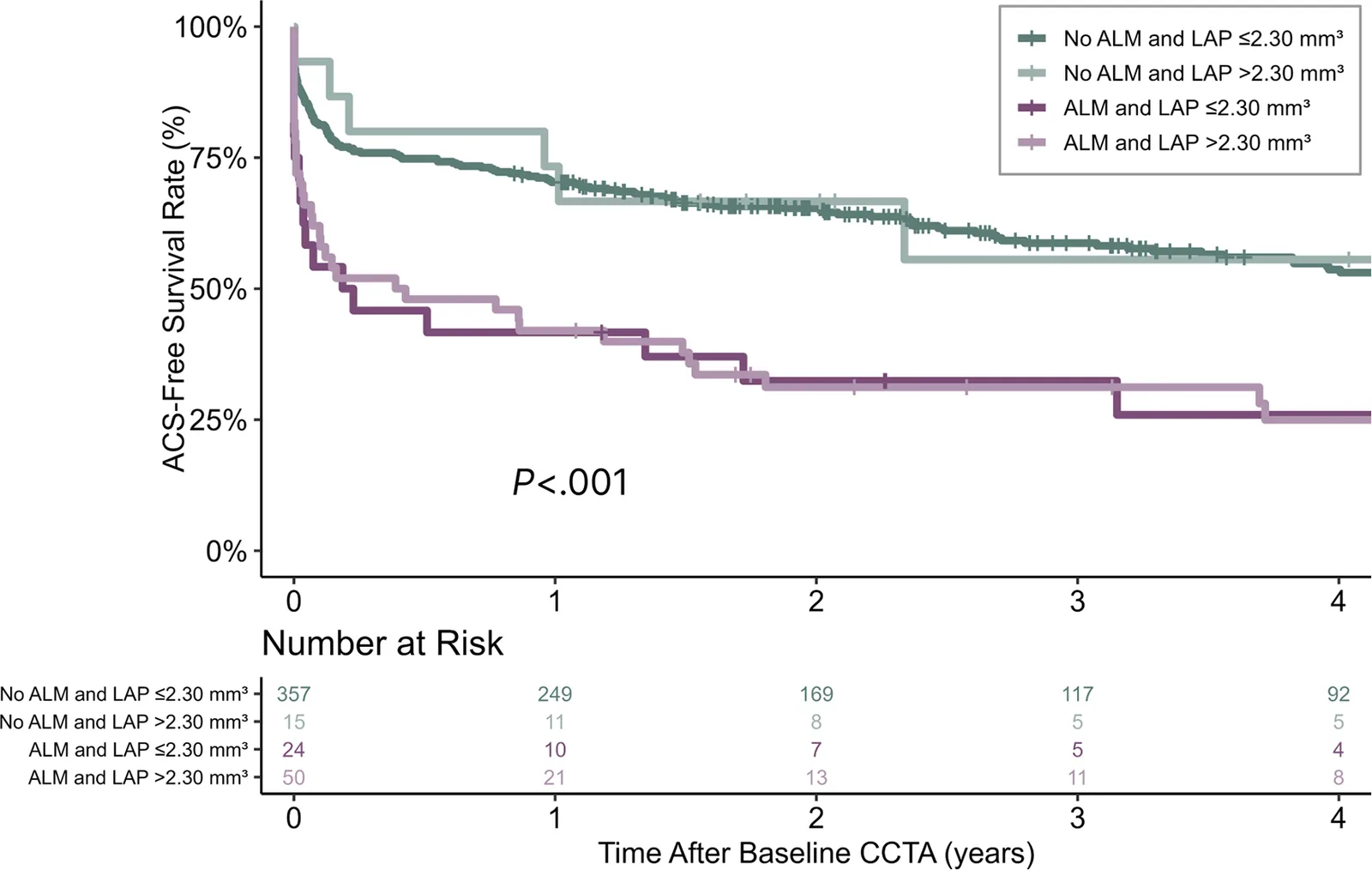
Graph shows Kaplan-Meier survival curves of the occurrence of acute coronary syndrome in cases and controls. Kaplan-Meier survival analysis demonstrates differences in acute coronary syndrome (ACS)–free survival among patients stratified by the presence of adverse lesion morphology (ALM) and low-attenuation noncalcified plaque (LAP) burden. LAP burden was dichotomized by the presence of a lesion with greater than 2.30 mm^3^ LAP. Regardless of LAP burden, patients with ALM had significantly lower ACS-free survival rates (log-rank *P* < .001). The number of patients at risk over time is shown below the x-axis. Tick marks indicate censored patients. ALM was defined as a lobular, bean, or spherical LAP shape with at least 180° degrees of embeddedness. CCTA = coronary CT angiography.

**Figure 4:**
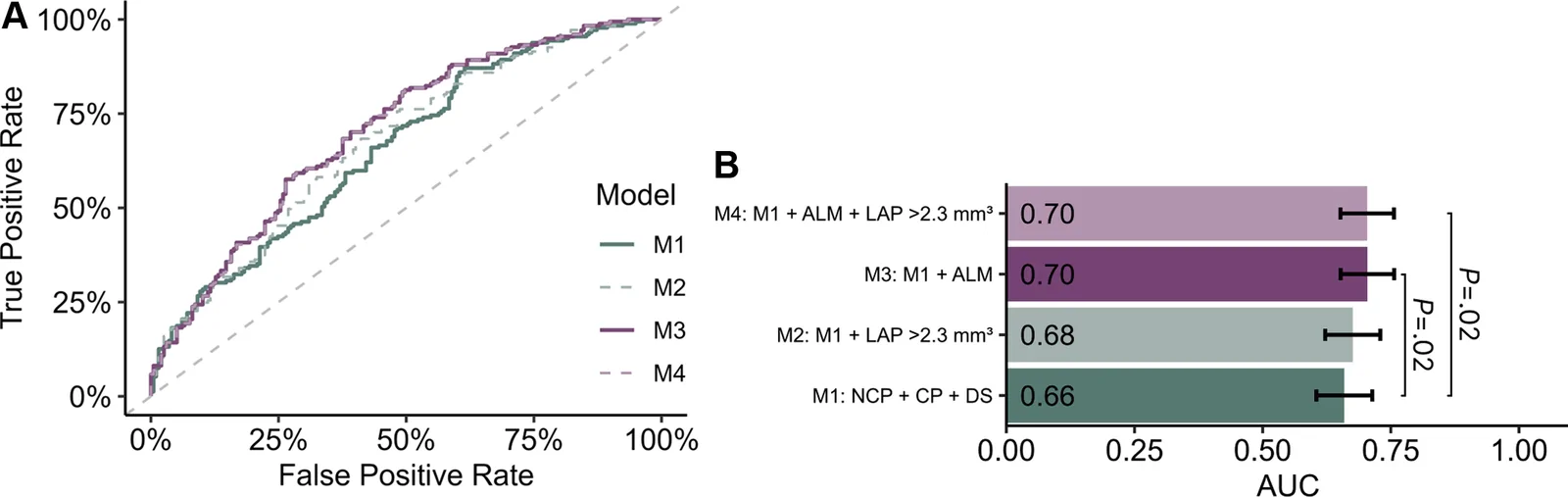
Graphs show time-dependent receiver operating characteristic curve analysis for acute coronary syndrome prediction. **(A)** Time-dependent receiver operating characteristic (ROC) curves at 2 years for the comparison of the performance of four Cox regression models for the prediction of acute coronary syndrome. Model 1 (M1) includes noncalcified plaque (NCP), calcified plaque (CP), and diameter stenosis (DS). Model 2 (M2) includes M1 variables plus lesion-level low-attenuation NCP (LAP) volume greater than 2.30 mm^3^, and model 3 (M3) includes M1 variables plus adverse LAP morphology (ALM; lobular, bean, or spherical LAP shape or LAP with ≥180° of embeddedness). Model 4 (M4) includes M1 variables plus both ALM and lesion-level LAP greater than 2.30 mm^3^. **(B)** The area under the receiver operating characteristic curve (AUC) for each model is shown with its 95% CI.

ACS-free survival rates were assessed by the presence of ALM and LAP volume (Fig 3). Four nested Cox regression models were compared. Model 1 included doubling of noncalcified and calcified plaque volume as well as diameter stenosis. Model 2 included the independent variables from model 1 and the presence of a lesion with a LAP volume greater than 2.30 mm^3^. Model 3 included the independent variables from model 1 and the presence of ALM. Model 4 included the independent variables from model 1 as well as the presence of ALM and the presence of a lesion with a LAP volume greater 2.30 mm^3^. ALM was significantly associated with ACS (model 4 adjusted hazard ratio [HR], 3.24 [95% CI: 1.44, 7.30]; *P* = .005) (Tables 3 and S5). The AUC value of the 2-year time-dependent receiver operator characteristic curve for each model was compared (Figs 4A, 4B). Compared with model 1 (AUC, 0.66 [95% CI: 0.60, 0.71]), models 3 and 4 demonstrated significantly higher discrimination for ACS (both AUC, 0.70 [95% CI: 0.65, 0.76]; *P* = .02). In contrast, model 2 (AUC, 0.68 [95% CI: 0.62, 0.73]; *P* = .28 vs model 1) did not provide incremental improvement. Univariable Cox regression analysis demonstrated that ALM was associated with culprit lesion precursors (HR, 7.25 [95% CI: 4.67, 11.30]; *P* < .001). However, after adjusting for LAP, noncalcified plaque, and calcified plaque volume, as well as diameter stenosis, ALM was not independently associated with culprit lesion precursors (adjusted HR, 1.73 [95% CI: 0.95, 3.16]; *P* = .08) (Table S6).

**Table 3:** Cox Regression Models Evaluating the Prognostic Value of Adverse LAP Morphology for Acute Coronary Syndrome

Characteristic	Univariable Analysis			Multivariable Analysis		
	HR (95% CI)	*P* Value	β (95% CI)	HR (95% CI)	*P* Value	β (95% CI)
ALM	3.92 (2.13, 7.21)	.001	1.37 (0.758, 1.98)	3.24 (1.44, 7.30)	.005	1.17 (0.362, 1.99)
LAP > 2.30 mm^3^	2.53 (1.39, 4.61)	.002	0.930 (0.332, 1.53)	0.98 (0.43, 2.21)	.96	−0.023 (−0.836, 0.791)
Noncalcified plaque volume (mm^3^)*	1.29 (1.10, 1.52)	.002	0.255 (0.094, 0.417)	1.12 (0.91, 1.38)	.30	0.111 (−0.098, 0.319)
Calcified plaque volume (mm^3^)*	1.03 (0.95, 1.12)	.50	0.029 (−0.055, 0.113)	0.89 (0.79, 1.00)	.05	−0.119 (−0.239, 0.001)
Diameter stenosis						
0%–24%	NA		NA	NA		NA
25%–49%	2.26 (1.27, 4.03)	.006	0.816 (0.240, 1.39)	2.70 (1.34, 5.44)	.006	0.992 (0.290, 1.69)
50%–69%	3.22 (1.60, 6.48)	.001	1.17 (0.472, 1.87)	3.43 (1.48, 7.96)	.004	1.23 (0.393, 2.07)
≥70%	4.64 (2.19, 9.83)	<.001	1.53 (0.784, 2.29)	3.38 (1.36, 8.41)	.009	1.22 (0.308, 2.13)
AIC				253.74		
C index				0.69		

Note.—Stratified Cox regression was performed using patient-level case-control matching identifiers, with robust variance estimation to account for clustering within matched pairs. AIC = Akaike information criterion, ALM = adverse low-attenuation noncalcified plaque morphology, C index = concordance index, LAP = low-attenuation noncalcified plaque, NA = not applicable, HR = hazard ratio.

* Per-doubling.

## Discussion

High-risk plaque features, such as the napkin-ring sign, positive remodeling, spotty calcification, and the presence of LAP have a strong association with future major adverse cardiac events. However, their use clinically remains limited by low positive predictive value, and the fact that many events still arise from plaques without high-risk features or from progression of initially non–high-risk plaques (5,27–30). In this secondary analysis of the ICONIC study, we evaluated whether qualitative LAP morphology, based on shape and DOE, improves the stratification of patients at risk for ACS. Morphologic features associated with ACS at survival analysis included lobular, bean, or spherical LAP shape and a LAP with a DOE of greater than or equal to 180° (log-rank *P* ≤ .001), which collectively defined ALM. Patients with ALM had more than a threefold increased adjusted risk of future ACS (adjusted HR, 3.24 [95% CI: 1.44, 7.30]; *P* = .005). Moreover, ALM significantly improved prediction of ACS beyond plaque burden and stenosis severity (AUC, 0.70 vs 0.66; *P* = .02), whereas the inclusion of LAP burden alone did not improve discrimination (AUC, 0.68 vs 0.66; *P* = .28).

The association between increased LAP burden and risk for adverse cardiac events has been well established in prior trials. Using a different quantitative CT platform, the primary ICONIC study found that patients with LAP had a 38% higher risk of ACS (adjusted HR, 1.38 [95% CI: 1.05, 1.81]; *P* = .02) (5). The Scottish Computed Tomography of the Heart (SCOT-HEART) trial (31) demonstrated that greater than 4% LAP burden showed increased risk for adverse events (unadjusted HR, 4.65 [95% CI: 2.06, 10.50]). Additional studies have demonstrated doubling of LAP burden was independently associated with increased risk for myocardial infarction or death (32,33). We used lesion-level LAP burden given its strong concordance with lipid-rich plaque at intravascular US and because a single high-LAP lesion may better represent vulnerable plaque than patient-level aggregate burden (23). In our study, univariable analysis showed that patients with a lesion that contained a more than 2.30 mm^3^ LAP had a greater risk for ACS (unadjusted HR, 2.53 [95% CI: 1.39, 4.61]; *P* = .002). However, after adjusting for plaque burden and diameter stenosis, this association was weakened (adjusted HR, 0.93 [95% CI: 0.33, 1.53]; *P* = .96). Differences in quantitative plaque software, study design (nested case-control vs randomized controlled trial), and multivariable models including noncalcified and calcified plaque volume may have attenuated the association of LAP burden with ACS risk. Although prior studies focused on quantitative measures of LAP burden, our findings suggest that the morphologic characteristics of LAP may provide additional discriminatory value beyond burden alone.

The conventional definition of high-risk plaque varies but typically combines the presence of low-attenuation plaque, the napkin-ring sign, spotty calcifications, and positive remodeling and shows an association with adverse events (5,27,34). However, high interobserver variability and low positive predictive value limit its widespread clinical application. In the former ICONIC study analysis, 39% of patients with high-risk plaque features were nonevent controls (5). Similarly, 94% of patients with high-risk plaque in the Prospective Multicenter Imaging Study for Evaluation of Chest Pain (PROMISE) trial did not experience adverse events during follow-up (27), whereas 62% of patients with low-attenuation plaque and positive remodeling in the SCOT-HEART trial were event free (34). In our study, however, the specificity and positive predictive value of ALM in stratifying patients with subsequent ACS was 92% and 77%, respectively. Importantly, ALM characteristics such as bean, lobular, or spherical LAP shape and a DOE of greater than or equal to 180° may represent a CT-equivalent finding of a vulnerable intraplaque necrotic core. Conversely, crescent-shaped LAP and a LAP with a DOE of less than 180° may represent pericoronary adipose tissue rather than vulnerable plaque. Because CCTA enables three-dimensional volumetric imaging, these findings, if substantiated in future studies, may confer a noninvasive and highly predictive definition of higher risk plaque that demonstrates increased risk for ACS. Notably, LAP shape was defined in three dimensions, whereas in prior studies with CCTA, histopathologic examination, intravascular US, and optical coherence tomography, LAP shape was based on two-dimensional definitions (11,35–37).

There are several limitations of the study. First, this was a secondary analysis of a retrospective nested case-control study, therefore, prospective validation is required. Second, although ICONIC included multiple centers and scanners, CT technology has advanced, and newer reconstruction algorithms may alter LAP appearance (38). Moreover, we used a single-validated AI-QCT platform (23), of which one reader was an executive of, which may introduce bias and limit generalizability (29). Although consensus review reduced the risk of bias, the reproducibility of ALM with contemporary scanners, different reconstruction techniques, and across different quantitative CT vendors needs further study. Third, overall agreement for ALM was substantial, but reproducibility within the four LAP morphologic shapes and DOE subtypes was lower, likely due to small sample sizes. Larger cohorts are needed to confirm the interobserver reproducibility of ALM. Fourth, our exclusion of patients with prior myocardial infarction or revascularization limits generalizability, and variation in downstream therapy may confound associations. Finally, the nested case-control design precludes longitudinal assessment in an all-comers population.

In conclusion, beyond conventional measures of plaque burden and diameter stenosis, ALM was independently associated with future ACS. Patients with ALM demonstrated a higher and earlier risk of ACS, as supported by positive predictive value and survival analyses. Thus, ALM may help identify patients and lesions at particularly high risk for future events, although its absence does not exclude vulnerability. Future investigations should aim to identify precursors to LAP to clarify the biologic progression of vulnerable plaque. Importantly, prior studies have shown that revascularization guided with high-risk plaque at invasive imaging reduced risk for major adverse cardiac events (39), suggesting that ALM could represent a noninvasive target for revascularization or intensified preventive therapy pending further validation.

## Supplemental Files

Appendices S1-S2, Tables S1-S6, Figures S1-S5

Conflicts of Interest
