# Supplementary material for: Association of Low-Attenuation Noncalcified Plaque Shape and Degree of Intraplaque Embeddedness Using Atherosclerotic Imaging–enabled Quantitative CT with Acute Coronary Syndrome
Source: Radiol Cardiothorac Imaging. 2026 Jul 23;8(4):e250117. doi: 10.1148/ryct.250117 (PMC13522894; doi:10.1148/ryct.250117)
Supplement: Supplementary file 1 [file ryct250117supp.pdf]

Supplemental materials undergo peer review but are not copyedited, and may include typographical errors.

©RSNA, 2026
10.1148/ryct.250117

APPENDIX S1: SUPPLEMENTAL METHODS

Clinical Events Adjudication Committee from ICONIC(5)

Fay Y. Lin, MD; Lohendran Bhaskaran, MD, Iksung Cho, MD; Ibrahim Danad, MD; Donghee Han, MD; Ji-Hyun Lee, MD; Joshua Schulman-Marcus, MD

Culprit Lesion Identification and Adjudication

At the time of ACS, a single culprit coronary lesion was adjudicated to baseline CCTA images using ICA in a subset of patients. Culprit lesion identification and adjudication was performed during the primary ICONIC study(1). Culprit lesions were first identified on ICA at the time of ACS. In patients with a single significant lesion on ICA, this lesion was identified as the culprit. In patients with more than one significant lesion, the culprit lesion was selected based on the most severe stenosis or complex lesion morphology, and assessment of the distribution of ischemia based on electrocardiogram findings. Ambiguous cases underwent consensus reading by a second cardiologist. Culprit lesion identification was performed blinded to CCTA imaging.

Once identified, culprit lesions were co-registered to lesions on CCTA by two readers, based on comparison of coronary segment location, distance from ostia, and location relative to coronary branch points. Readers were unblinded from ICA and CCTA images to allow culprit lesion adjudication.

Culprit lesions could not be adjudicated in 104 patients with ACS due to no ICA being performed, missing ICA images, or no culprit lesion identified on ICA. Thus, a subset of 121 patients with culprit lesion adjudication were included for lesion-level within-patient analyses. Culprit lesions were adjudicated in 121 patients at the time of ACS. Within these patients, there were 121 culprit lesions and 829 non-culprit lesions.

Lesion-Level Statistical Analysis

For odds ratios within-patients, culprit lesions were compared with non-culprit lesions using mixed logistic regression, using the patient identifier as a random effect. Cox regression analysis was performed using time to first ACS event, or event-free follow-up date. The robust variance estimator was used to adjust for within-patient correlation for lesion-level analysis.

APPENDIX S2: SUPPLEMENTAL RESULTS

Association of ALM with Culprit Lesion Precursors

At the lesion level, the association of ALM with development into culprit ACS-causing lesions was also assessed in a subgroup analysis. Only 121/223 (54%) of patients who experienced ACS underwent culprit lesion adjudication. In total, 121 culprit lesions were compared with 829 non-culprit lesions. ALM was present in 50/950 (5%) of lesions, 28/121 (23%) within culprit lesions and 22/829 (3%) within non-culprit lesions (**Tables S3b** and S**4**; **Fig S5**).

Supplemental materials undergo peer review but are not copyedited, and may include typographical errors.

SUPPLEMENTAL TABLES

Table S1: Low Attenuation Non-Calcified Plaque Geometric Shape Categories

Shape	Definition
Crescent	A thin, smooth, arc-like deposit with tapered width that partially curves along the contour of the vessel.
Lobular	An irregularly contoured plaque with multiple rounded extensions or protrusions.
Sphere	A compact, round or ovoid deposit with smooth, symmetrical margins.
Bean	An elongated, curved, oblong, and asymmetric plaque with one flatter edge and one rounded side.

Supplemental materials undergo peer review but are not copyedited, and may include typographical errors.

Table S2: Additional Atherosclerotic Plaque Characteristics from Coronary CT Angiography and Association with Acute Coronary Syndrome

Variable	Overall N = 446	ACS (Cases) N = 223	Non-ACS (Controls) N = 223	Odds Ratio	
				OR [95% CI]	*P* value
Percent Atheroma Volume (%)	21.50 [16.27, 28.90]	22.47 [16.89, 29.56]	20.83 [15.29, 28.49]	1.01 [0.99, 1.03]	.27
Low-Attenuation Non-Calcified Plaque (%)	0.00 [0.00, 0.10]	0.00 [0.00, 0.35]	0.00 [0.00, 0.02]	1.32 [1.06, 1.63]	.01
Non-Calcified Plaque (%)	73.53 [57.55, 91.09]	75.52 [59.48, 90.50]	72.36 [56.04, 92.13]	1.01 [1.00, 1.01]	.19
Total Calcified Plaque (%)	26.18 [7.56, 42.34]	24.25 [7.56, 40.67]	27.59 [7.54, 42.94]	0.99 [0.99, 1.00]	.20
LAP >0 mm^3 and RI >1.1	152/446 (34.08%)	90/223 (40.36%)	62/223 (27.80%)	1.76 [1.18, 2.61]	.005
Maximum Diameter Stenosis					
0	6/446 (1.35%)	3/223 (1.35%)	3/223 (1.35%)	—	
1-24	152/446 (34.08%)	62/223 (27.80%)	90/223 (40.36%)	0.69 [0.13, 3.53]	.65
25-49	169/446 (37.89%)	84/223 (37.67%)	85/223 (38.12%)	0.99 [0.19, 5.04]	.99
50-69	71/446 (15.92%)	45/223 (20.18%)	26/223 (11.66%)	1.73 [0.33, 9.21]	.52
≥70	48/446 (10.76%)	29/223 (13.00%)	19/223 (8.52%)	1.53 [0.28, 8.37]	.63
Crescent LAP Shape	50/446 (11.21%)	29/223 (13.00%)	21/223 (9.42%)	1.44 [0.79, 2.61]	.23
Lobular LAP Shape	44/446 (9.87%)	31/223 (13.90%)	13/223 (5.83%)	2.61 [1.33, 5.13]	.005
Spherical LAP Shape	15/446 (3.36%)	15/223 (6.73%)	0/223 (0.00%)	-	-
Bean LAP Shape	54/446 (12.11%)	43/223 (19.28%)	11/223 (4.93%)	4.60 [2.31, 9.19]	<.001
<90 LAP DOE	9/446 (2.02%)	5/223 (2.24%)	4/223 (1.79%)	1.26 [0.33, 4.74]	.74
90-179 LAP DOE	66/446 (14.80%)	41/223 (18.39%)	25/223 (11.21%)	1.78 [1.04, 3.05]	.03
180-269 LAP DOE	53/446 (11.88%)	43/223 (19.28%)	10/223 (4.48%)	5.09 [2.49, 10.4]	<.001
270-360 LAP DOE	25/446 (5.61%)	21/223 (9.42%)	4/223 (1.79%)	5.69 [1.92, 16.9]	.002

[1] Median [Q1, Q3]; n/N (%)

Abbreviations: ACS, acute coronary syndrome; OR, odds ratio; ; LAP, low-attenuation non-calcified plaque; ALM, adverse LAP morphology; DOE, degree of intra-plaque embeddedness.

Supplemental materials undergo peer review but are not copyedited, and may include typographical errors.

Table S3: Lesion- and Patient-Level Diagnostic Performance of LAP Morphologic Features. (a) Patient-level statistics assessed the diagnostic performance for identifying patients with subsequent acute coronary syndrome. (b) Lesion-level statistics were performed to determine the diagnostic performance for identifying culprit lesion precursors.

**(a) Patient-Level**	N	Sensitivity [95% CI]	Specificity [95% CI]	PPV [95% CI]	NPV [95% CI]	Accuracy [95% CI]
DOE <90°	9	2.24% [0.96, 5.14%]	98.21% [95.48, 99.30%]	55.56% [26.67, 81.12%]	50.11% [45.45, 54.78%]	50.22% [45.60, 54.84%]
DOE 90-179°	66	18.39% [13.85, 23.99%]	88.79% [83.97, 92.29%]	62.12% [50.06, 72.85%]	52.11% [47.09, 57.08%]	53.59% [48.95, 58.17%]
DOE 180-269°	53	19.28% [14.64, 24.96%]	95.52% [91.94, 97.55%]	81.13% [68.64, 89.41%]	54.20% [49.26, 59.06%]	57.40% [52.77, 61.91%]
DOE 270-360°	25	9.42% [6.24, 13.97%]	98.21% [95.48, 99.30%]	84.00% [65.35, 93.60%]	52.02% [47.25, 56.75%]	53.81% [49.17, 58.39%]
Crescent Shape	50	13.00% [9.21, 18.05%]	90.58% [86.03, 93.76%]	58.00% [44.23, 70.62%]	51.01% [46.10, 55.90%]	51.79% [47.16, 56.40%]
Lobular Shape	44	13.90% [9.97, 19.06%]	94.17% [90.28, 96.56%]	70.45% [55.78, 81.84%]	52.24% [47.36, 57.08%]	54.04% [49.40, 58.61%]
Bean Shape	54	19.28% [14.64, 24.96%]	95.07% [91.38, 97.22%]	79.63% [67.10, 88.23%]	54.08% [49.13, 58.95%]	57.17% [52.54, 61.69%]
Sphere Shape	15	6.73% [4.12, 10.80%]	100.00% [98.31, 100.00%]	100.00% [79.61, 100.00%]	51.74% [47.03, 56.42%]	53.36% [48.72, 57.94%]
LAP >2.30 mm³	65	20.63% [15.84, 26.41%]	91.48% [87.08, 94.48%]	70.77% [58.80, 80.42%]	53.54% [48.52, 58.49%]	56.05% [51.42, 60.59%]
LAP >0 mm³	157	42.60% [36.29, 49.16%]	72.20% [65.98, 77.66%]	60.51% [52.70, 67.82%]	55.71% [49.94, 61.32%]	57.40% [52.77, 61.91%]
ALM	74	25.56% [20.28, 31.67%]	92.38% [88.13, 95.19%]	77.03% [66.25, 85.13%]	55.38% [50.30, 60.35%]	58.97% [54.35, 63.44%]
ALM and LAP >2.30 mm³	50	17.49% [13.07, 23.01%]	95.07% [91.38, 97.22%]	78.00% [64.76, 87.25%]	53.54% [48.61, 58.39%]	56.28% [51.64, 60.81%]

**(b) Lesion-Level**	N	Sensitivity [95% CI]	Specificity [95% CI]	PPV [95% CI]	NPV [95% CI]	Accuracy [95% CI]
DOE <90°	3	0.00% [0.00, 3.08%]	99.64% [98.94, 99.88%]	0.00% [0.00, 56.15%]	87.22% [84.95, 89.20%]	86.95% [84.66, 88.94%]
DOE 90-179°	31	9.09% [5.15, 15.55%]	97.59% [96.30, 98.43%]	35.48% [21.12, 53.05%]	88.03% [85.77, 89.97%]	86.32% [83.98, 88.36%]
DOE 180-269°	30	16.53% [10.96, 24.16%]	98.79% [97.79, 99.34%]	66.67% [48.78, 80.77%]	89.02% [86.84, 90.88%]	88.32% [86.12, 90.21%]
DOE 270-360°	15	9.09% [5.15, 15.55%]	99.52% [98.77, 99.81%]	73.33% [48.05, 89.10%]	88.24% [86.01, 90.15%]	88.00% [85.78, 89.91%]
Crescent Shape	20	2.48% [0.85, 7.04%]	97.95% [96.74, 98.72%]	15.00% [5.24, 36.04%]	87.31% [85.02, 89.30%]	85.79% [83.42, 87.87%]
Lobular Shape	21	10.74% [6.39, 17.52%]	99.03% [98.11, 99.51%]	61.90% [40.88, 79.25%]	88.37% [86.15, 90.28%]	87.79% [85.55, 89.72%]
Bean Shape	29	14.88% [9.62, 22.29%]	98.67% [97.64, 99.26%]	62.07% [44.00, 77.31%]	88.82% [86.62, 90.69%]	88.00% [85.78, 89.91%]
Sphere Shape	13	7.44% [3.96, 13.53%]	99.52% [98.77, 99.81%]	69.23% [42.37, 87.32%]	88.05% [85.81, 89.97%]	87.79% [85.55, 89.72%]
LAP >2.30 mm³	37	16.53% [10.96, 24.16%]	97.95% [96.74, 98.72%]	54.05% [38.38, 68.96%]	88.94% [86.74, 90.81%]	87.58% [85.33, 89.53%]
LAP >0 mm³	100	34.71% [26.82, 43.55%]	93.00% [91.06, 94.55%]	42.00% [32.80, 51.79%]	90.71% [88.57, 92.48%]	85.58% [83.20, 87.67%]
ALM	50	23.14% [16.52, 31.41%]	97.35% [96.01, 98.24%]	56.00% [42.31, 68.84%]	89.67% [87.51, 91.49%]	87.89% [85.67, 89.82%]
ALM and LAP >2.30 mm³	27	14.05% [8.96, 21.35%]	98.79% [97.79, 99.34%]	62.96% [44.23, 78.47%]	88.73% [86.53, 90.61%]	88.00% [85.78, 89.91%]

Supplemental materials undergo peer review but are not copyedited, and may include typographical errors.

Table S4: Within-Patient Lesion-Level Atherosclerotic Plaque Characteristics from Coronary CT Angiography

	Within-Patient Lesion-Level Comparison			Odds Ratio	
**Variable**	**Overall** N = 950[1]	**Culprit** N = 121[1]	**Non-Culprit** N = 829[1]	**OR [95% CI]**	**p-value**
Total Plaque Volume (mm³)	9.40 [3.50-37.80]	104.00 [24.60-227.70]	7.70 [3.20-25.90]	1.01 [1.00, 1.01]	p<0.001
Low Density Plaque Volume (mm³)	0.00 [0.00-0.00]	0.00 [0.00-0.60]	0.00 [0.00-0.00]	1.14 [1.06, 1.23]	p<0.001
Non-Calcified Plaque Volume (mm³)	7.10 [2.90-27.00]	52.90 [16.70-150.80]	5.90 [2.60-17.20]	1.01 [1.01, 1.01]	p<0.001
Total Calcified Plaque Volume (mm³)	0.90 [0.00-9.20]	18.60 [1.30-76.90]	0.60 [0.00-5.70]	1.01 [1.01, 1.01]	p<0.001
Percent Atheroma Volume (%)	19.71 [13.16-32.01]	38.11 [23.80-46.21]	18.77 [12.57-28.25]	1.06 [1.05, 1.07]	p<0.001
Low Density Plaque (%)	0.00 [0.00-0.00]	0.00 [0.00-0.34]	0.00 [0.00-0.00]	1.09 [1.01, 1.18]	p=0.033
Non-Calcified Plaque (%)	86.32 [61.29-100.00]	71.51 [54.26-90.91]	87.50 [62.82-100.00]	0.99 [0.98, 0.99]	p<0.001
Total Calcified Plaque (%)	13.32 [0.00-38.46]	27.14 [7.89-45.16]	11.54 [0.00-36.07]	1.01 [1.01, 1.02]	p<0.001
Minimum Lumen Diameter (mm)	2.32 [1.84-2.93]	2.24 [1.82-2.84]	2.32 [1.85-2.94]	1.00 [0.80, 1.26]	p=0.981
Remodeling Index	1.20 [1.10-1.30]	1.30 [1.20-1.50]	1.20 [1.10-1.30]	22.8 [9.22, 56.3]	p<0.001
Lesion Length (mm)	7.75 [4.25-18.75]	31.00 [14.00-58.50]	6.75 [4.25-14.25]	1.03 [1.03, 1.04]	p<0.001
Diameter Stenosis (%)	10.00 [4.00-23.00]	32.00 [17.00-52.00]	9.00 [3.00-19.00]	1.05 [1.04, 1.06]	p<0.001
LAP >0 mm³ and RI >1.1	95/950 (10.00%)	39/121 (32.23%)	56/829 (6.76%)	6.57 [4.11, 10.5]	p<0.001
LAP >2.30 mm³	37/950 (3.89%)	20/121 (16.53%)	17/829 (2.05%)	9.46 [4.80, 18.6]	p<0.001
LAP >0 mm³	100/950 (10.53%)	42/121 (34.71%)	58/829 (7.00%)	7.07 [4.46, 11.2]	p<0.001
Diameter Stenosis					
0	174/950 (18.32%)	9/121 (7.44%)	165/829 (19.90%)	—	
1-24	565/950 (59.47%)	41/121 (33.88%)	524/829 (63.21%)	1.43 [0.68, 3.01]	p=0.341
25-49	153/950 (16.11%)	40/121 (33.06%)	113/829 (13.63%)	6.49 [3.03, 13.9]	p<0.001
50-69	39/950 (4.11%)	23/121 (19.01%)	16/829 (1.93%)	26.4 [10.4, 66.5]	p<0.001
≥70	19/950 (2.00%)	8/121 (6.61%)	11/829 (1.33%)	13.3 [4.30, 41.3]	p<0.001
LAP Morphologic Features					
None	900/950 (94.74%)	93/121 (76.86%)	807/829 (97.35%)	—	
Bean, Lobular, or Sphere Shaped LAP **or** ≥180º DOE	14/950 (1.47%)	6/121 (4.96%)	8/829 (0.97%)	6.51 [2.21, 19.2]	p<0.001
Bean, Lobular, or Sphere Shaped LAP **and** ≥180º DOE	36/950 (3.79%)	22/121 (18.18%)	14/829 (1.69%)	13.6 [6.75, 27.6]	p<0.001
ALM and LAP					
No ALM and LAP ≤2.30 mm³	890/950 (93.68%)	90/121 (74.38%)	800/829 (96.50%)	—	
No ALM and LAP >2.30 mm³	10/950 (1.05%)	3/121 (2.48%)	7/829 (0.84%)	3.81 [0.97, 15.0]	p=0.056
ALM and LAP ≤2.30 mm³	23/950 (2.42%)	11/121 (9.09%)	12/829 (1.45%)	8.15 [3.49, 19.0]	p<0.001
ALM and LAP >2.30 mm³	27/950 (2.84%)	17/121 (14.05%)	10/829 (1.21%)	15.1 [6.72, 34.0]	p<0.001
Crescent LAP Shape	20/950 (2.11%)	3/121 (2.48%)	17/829 (2.05%)	1.21 [0.35, 4.21]	p=0.759
Lobular LAP Shape	21/950 (2.21%)	13/121 (10.74%)	8/829 (0.97%)	12.4 [5.01, 30.5]	p<0.001
Sphere LAP Shape	13/950 (1.37%)	9/121 (7.44%)	4/829 (0.48%)	16.6 [5.02, 54.7]	p<0.001
Bean LAP Shape	29/950 (3.05%)	18/121 (14.88%)	11/829 (1.33%)	13.0 [5.97, 28.3]	p<0.001
<90 LAP DOE	3/950 (0.32%)	0/121 (0.00%)	3/829 (0.36%)	0.00 [0.00, Inf]	p>0.999
90-179 LAP DOE	31/950 (3.26%)	11/121 (9.09%)	20/829 (2.41%)	4.04 [1.89, 8.67]	p<0.001
180-269 LAP DOE	30/950 (3.16%)	20/121 (16.53%)	10/829 (1.21%)	16.2 [7.38, 35.6]	p<0.001
270-360 LAP DOE	15/950 (1.58%)	11/121 (9.09%)	4/829 (0.48%)	20.6 [6.46, 65.9]	p<0.001
Vessel Territory					
LAD	318/950 (33.47%)	69/121 (57.02%)	249/829 (30.04%)	—	
LCX	203/950 (21.37%)	18/121 (14.88%)	185/829 (22.32%)	0.35 [0.20, 0.61]	p<0.001
RCA	429/950 (45.16%)	34/121 (28.10%)	395/829 (47.65%)	0.31 [0.20, 0.48]	p<0.001

[1] Median [Q1-Q3]; n/N (%)

Abbreviations: CI = Confidence Interval, OR = Odds Ratio

Supplemental materials undergo peer review but are not copyedited, and may include typographical errors.

Table S5: Nested Cox Regression Models Evaluating the Incremental Prognostic Value of Adverse LAP Morphology for Acute Coronary Syndrome. Nested multivariable Cox regression models evaluating the association of adverse low attenuation non-calcified plaque (LAP) morphology with acute coronary syndrome. Models adjust for non-calcified plaque volume (per doubling), calcified plaque volume (per doubling), diameter stenosis, and the presence of a lesion with >2.30 mm^3. Stratified Cox regression was performed using patient-level case-control matching identifiers, with robust variance estimation to account for clustering within matched pairs.

	Univariable		Model 1		Model 2		Model 3		Model 4	
**Characteristic**	**HR [95% CI]**	**p-value**	**HR [95% CI]**	**p-value**	**HR [95% CI]**	**p-value**	**HR [95% CI]**	**p-value**	**HR [95% CI]**	**p-value**
ALM	3.92 [2.13, 7.21]	p<0.001					3.20 [1.58, 6.48]	p=0.001	3.24 [1.44, 7.30]	p=0.005
Non-Calcified Plaque Volume (mm³)[1]	1.29 [1.10, 1.52]	p=0.002	1.24 [1.01, 1.53]	p=0.037	1.17 [0.96, 1.44]	p=0.126	1.12 [0.91, 1.37]	p=0.296	1.12 [0.91, 1.38]	p=0.299
Calcified Plaque Volume (mm³)[1]	1.03 [0.95, 1.12]	p=0.496	0.87 [0.77, 0.98]	p=0.023	0.88 [0.78, 0.99]	p=0.031	0.89 [0.79, 1.00]	p=0.052	0.89 [0.79, 1.00]	p=0.052
Diameter Stenosis										
0-24	—		—		—		—		—	
25-49	2.26 [1.27, 4.03]	p=0.006	2.48 [1.21, 5.05]	p=0.013	2.58 [1.28, 5.19]	p=0.008	2.70 [1.34, 5.45]	p=0.006	2.70 [1.34, 5.44]	p=0.006
50-69	3.22 [1.60, 6.48]	p=0.001	3.54 [1.53, 8.17]	p=0.003	3.52 [1.54, 8.06]	p=0.003	3.44 [1.48, 7.97]	p=0.004	3.43 [1.48, 7.96]	p=0.004
≥70	4.64 [2.19, 9.83]	p<0.001	4.51 [1.80, 11.3]	p=0.001	4.26 [1.70, 10.7]	p=0.002	3.39 [1.36, 8.44]	p=0.009	3.38 [1.36, 8.41]	p=0.009
LAP >2.30 mm³	2.53 [1.39, 4.61]	p=0.002			1.90 [0.96, 3.77]	p=0.067			0.98 [0.43, 2.21]	p=0.957
AIC			262.18		260.43		251.74		253.74	
C-Index			0.65		0.65		0.69		0.69	

[1] Per-doubling

Abbreviations: CI = Confidence Interval, HR = Hazard Ratio, NA

Supplemental materials undergo peer review but are not copyedited, and may include typographical errors.

Table S6: Nested Cox Regression Models Evaluating Lesion-Level Predictors of Culprit Lesion Status Among Patients with Acute Coronary Syndrome. Nested multivariable Cox regression models evaluating the association of adverse low attenuation non-calcified plaque (LAP) morphology with culprit lesion status among patients with acute coronary syndrome (ACS). Models were restricted to patients with ACS and compare culprit versus non-culprit lesions. All models adjust for lesion-level non-calcified plaque volume (per doubling), calcified plaque volume (per doubling), and diameter stenosis. Additional variables include LAP >2.30 mm^3 and/or adverse lesion morphology (ALM). Cox regression was performed with robust variance estimation to account for clustering of lesions within patients.

	Univariable		Model 1		Model 2		Model 3		Model 4	
**Characteristic**	**HR [95% CI]**	**p-value**	**HR [95% CI]**	**p-value**	**HR [95% CI]**	**p-value**	**HR [95% CI]**	**p-value**	**HR [95% CI]**	**p-value**
ALM	7.25 [4.67, 11.3]	p<0.001					1.55 [0.86, 2.80]	p=0.142	1.73 [0.95, 3.16]	p=0.076
Non-Calcified Plaque Volume (mm^3)[1]	1.59 [1.46, 1.73]	p<0.001	1.30 [1.12, 1.52]	p<0.001	1.29 [1.11, 1.51]	p=0.001	1.25 [1.07, 1.45]	p=0.005	1.26 [1.08, 1.47]	p=0.004
Calcified Plaque Volume (mm^3)[1]	1.39 [1.30, 1.48]	p<0.001	1.02 [0.90, 1.16]	p=0.725	1.02 [0.91, 1.16]	p=0.695	1.04 [0.92, 1.17]	p=0.549	1.03 [0.92, 1.16]	p=0.589
Diameter Stenosis										
0-24	—		—		—		—		—	
25-49	5.66 [3.66, 8.75]	p<0.001	2.72 [1.55, 4.75]	p<0.001	2.72 [1.55, 4.77]	p<0.001	2.71 [1.55, 4.71]	p<0.001	2.67 [1.52, 4.70]	p<0.001
50-69	11.6 [6.64, 20.3]	p<0.001	4.17 [2.01, 8.64]	p<0.001	4.09 [1.91, 8.74]	p<0.001	3.82 [1.81, 8.04]	p<0.001	3.98 [1.85, 8.60]	p<0.001
≥70	17.2 [8.13, 36.6]	p<0.001	6.10 [2.53, 14.7]	p<0.001	6.01 [2.43, 14.8]	p<0.001	5.46 [2.32, 12.8]	p<0.001	5.58 [2.30, 13.6]	p<0.001
LAP >2.30 mm^3	5.98 [3.74, 9.55]	p<0.001			1.07 [0.51, 2.26]	p=0.856			0.79 [0.35, 1.79]	p=0.574
AIC			1,274.94		1,276.88		1,274.20		1,275.71	
C-Index			0.84		0.84		0.84		0.84	
[1] Per-doubling										
Abbreviations: CI = Confidence Interval, HR = Hazard Ratio, NA										

Supplemental materials undergo peer review but are not copyedited, and may include typographical errors.

SUPPLEMENTAL FIGURES

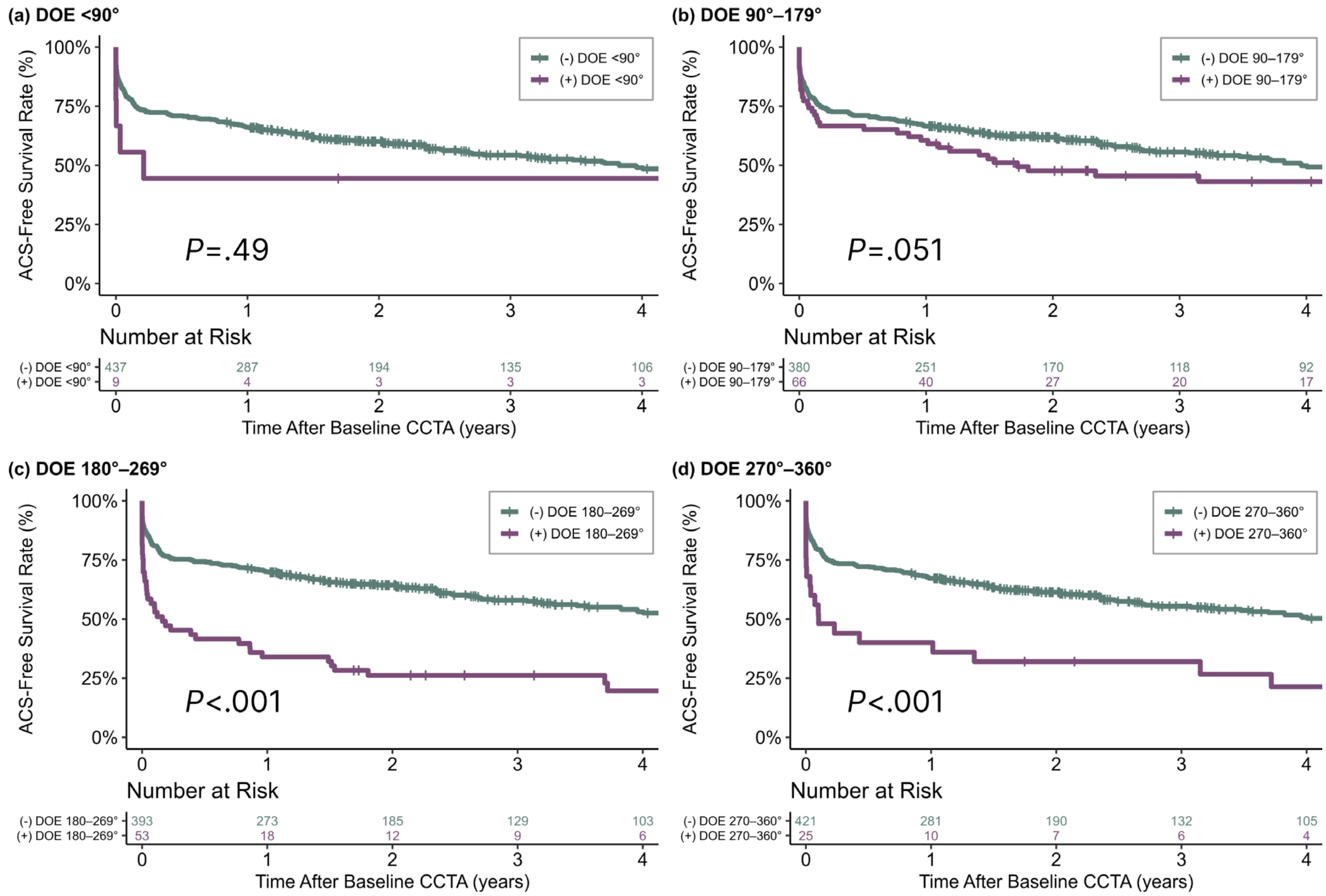

Figure S1: Kaplan-Meier Curves of ACS-Free Survival Stratified by Degree of Embeddedness (DOE) of Low Attenuation Non-Calcified Plaque

Kaplan-Meier survival analysis depicting acute coronary syndrome (ACS)-free survival by degree of embeddedness (DOE) of low attenuation non-calcified plaque (LAP) on coronary CT angiography (CCTA). Embeddedness was categorized into four groups: (a) <90°, (b) 90–179°, (c) 180–269°, and (d) 270–360°. Patients with embedded plaque (purple line) were compared to those without embedded plaque (green line) within each angular category. Increasing DOE was associated with significantly reduced ACS-free survival, particularly in the 180–269° and 270–360° groups (p<0.001), suggesting greater embeddedness confers higher risk of future ACS. The number at risk is shown below each time point.

Supplemental materials undergo peer review but are not copyedited, and may include typographical errors.

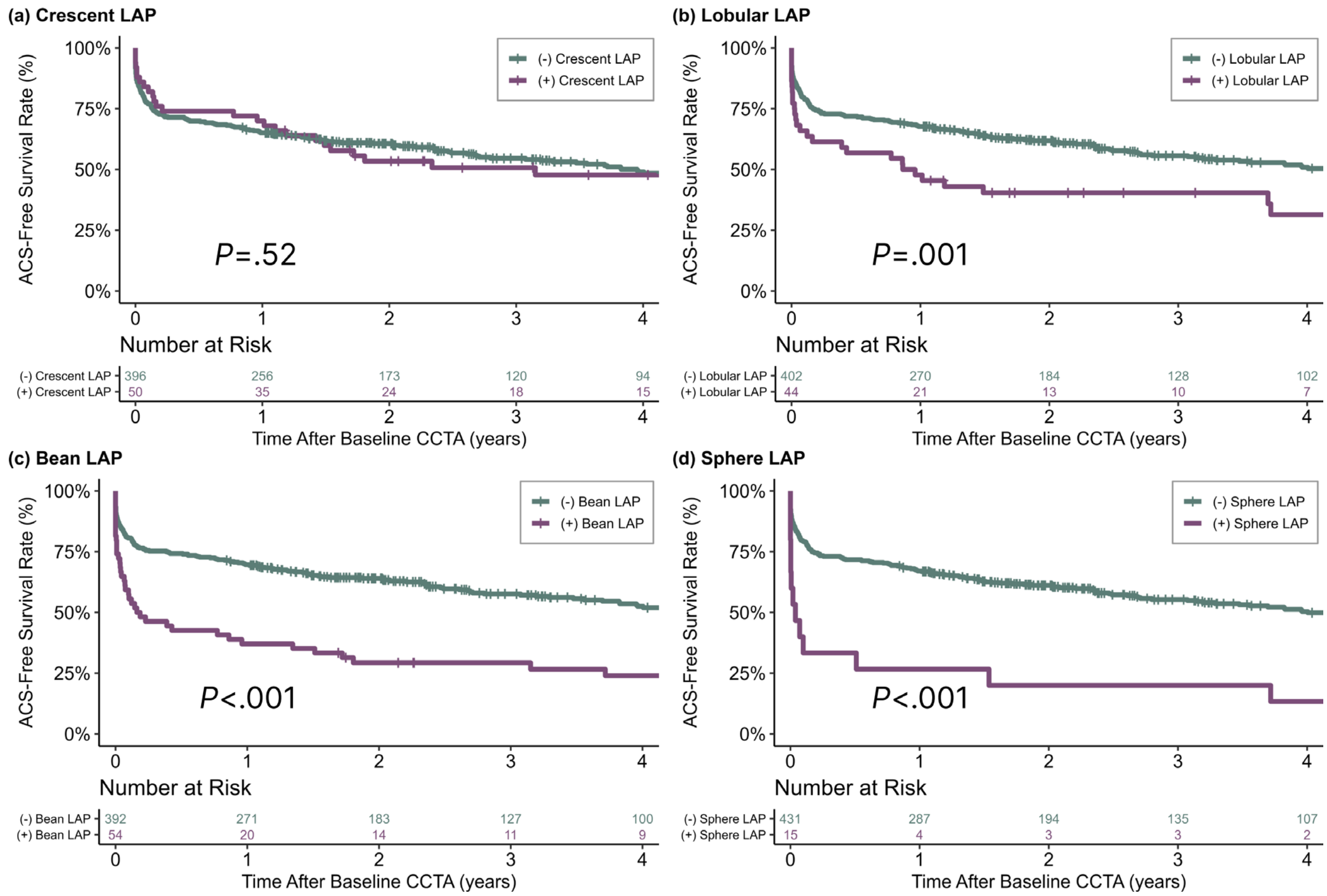

Figure S2: **Kaplan-Meier Curves of ACS-Free Survival Stratified by Geometric Shape of Low Attenuation Non-Calcified Plaque (LAP)**

Kaplan-Meier survival analysis evaluating acute coronary syndrome (ACS)-free survival based on the geometric shape of low attenuation non-calcified plaque (LAP) on coronary CT angiography (CCTA). LAP morphology was categorized into four distinct shapes: (a) crescent, (b) lobular, (c) bean, and (d) sphere. Patients with the respective LAP shape (purple line) were compared to those without that shape (green line). Lobular, bean, and sphere morphologies were each associated with significantly reduced ACS-free survival ($p \leq 0.001$), while crescent-shaped LAP showed no significant association ($p = 0.522$). The number at risk is shown for each group over time.

Supplemental materials undergo peer review but are not copyedited, and may include typographical errors.

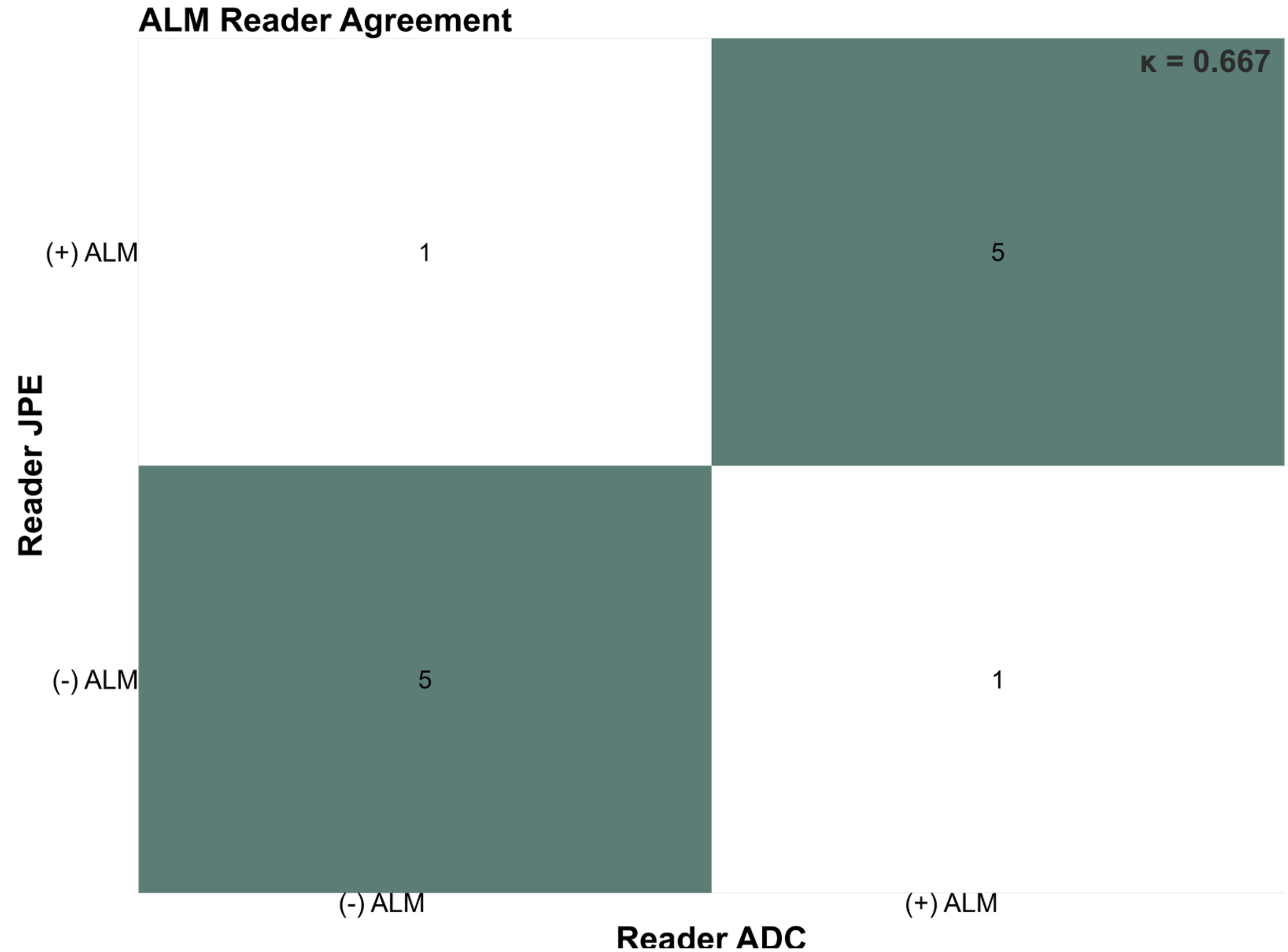

Figure S3: Reader Agreement of Adverse Low Attenuation Non-Calcified Plaque Morphology (ALM).

Cohen's kappa is annotated in the top right corner.

Supplemental materials undergo peer review but are not copyedited, and may include typographical errors.

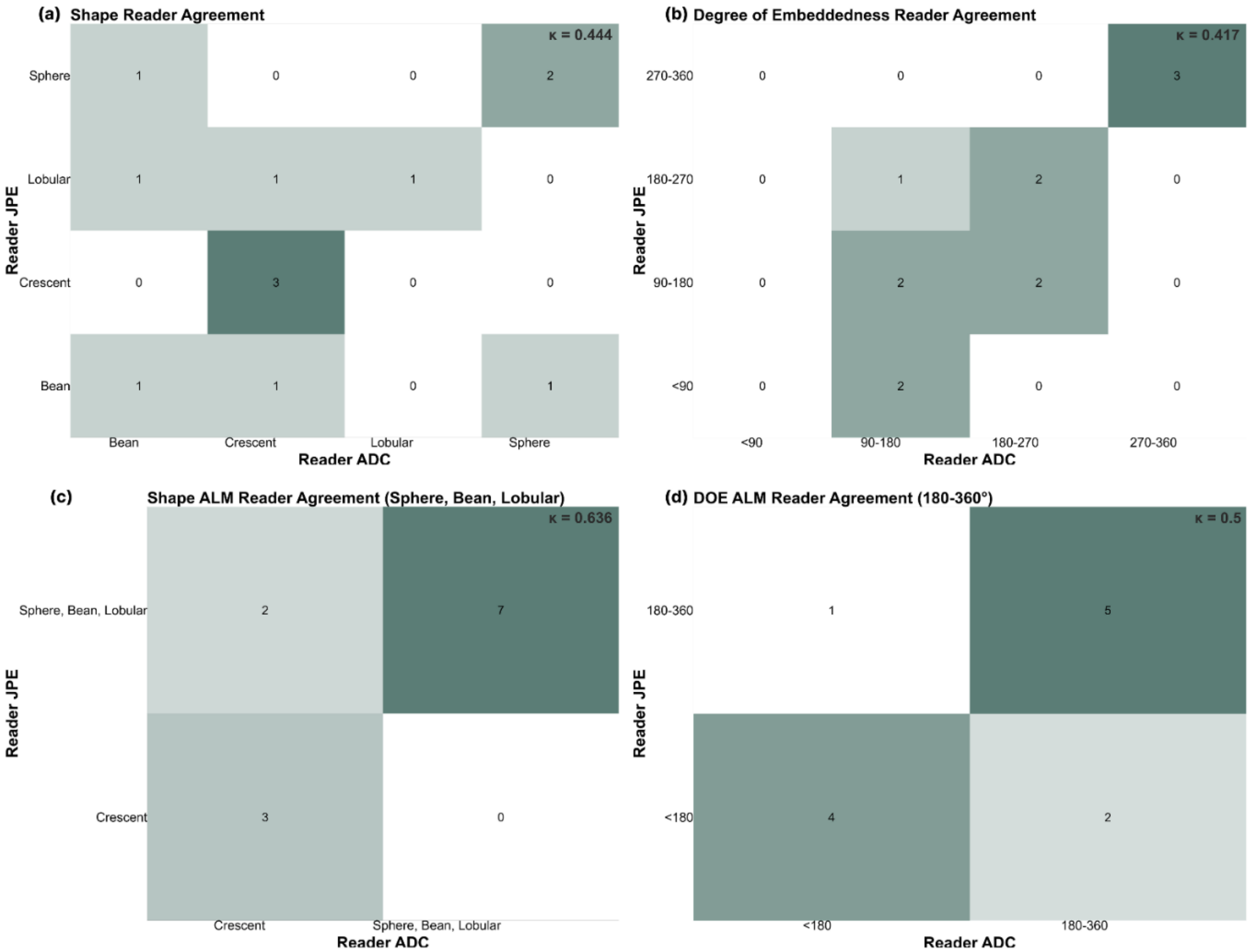

Figure S4: Reader Agreement of Low Attenuation Non-Calcified Plaque (a) Shape, (b) Degree of Embeddedness, (c) Adverse LAP Morphology Shapes, and (d) Adverse LAP Morphology Degree of Embeddedness.

Cohen's kappa is annotated in the top right corner.

Supplemental materials undergo peer review but are not copyedited, and may include typographical errors.

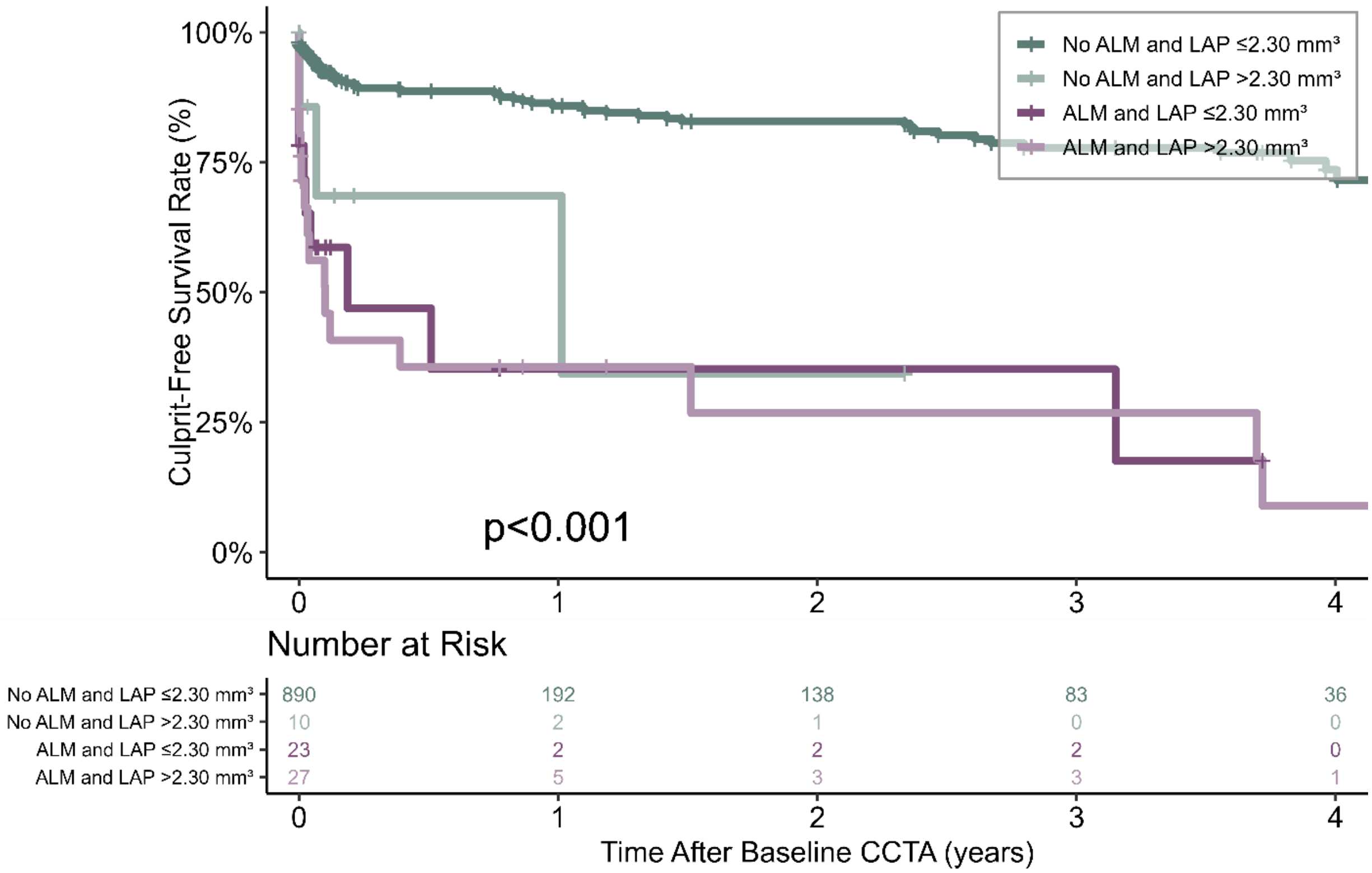

Figure S5: **Kaplan-Meier Curves of Culprit-Free Survival Stratified by Adverse Low Attenuation Plaque Morphology (ALM) and Low Attenuation Non-Calcified Plaque (LAP) Burden**

Kaplan-Meier survival analysis evaluating culprit-free survival was assessed in 950 lesions from 121 patients who experienced acute coronary syndrome and underwent culprit lesion adjudication. The number at risk at each time point is displayed below each plot.

ALM = Adverse low attenuation non-calcified plaque morphology (lobular, bean, or sphere shaped low attenuation non-calcified plaque or low attenuation non-calcified plaque with ≥180º degrees of embeddedness)
LAP = Low attenuation non-calcified plaque
